# Adult Cochlear Implant Recipients’ Perspectives on Experiences With Music in Everyday Life: A Multifaceted and Dynamic Phenomenon

**DOI:** 10.3389/fnins.2019.01229

**Published:** 2019-11-21

**Authors:** Kate Gfeller, Virginia Driscoll, Adam Schwalje

**Affiliations:** ^1^School of Music, College of Liberal Arts and Sciences, The University of Iowa, Iowa City, IA, United States; ^2^Department of Communication Sciences and Disorders, College of Liberal Arts and Sciences, The University of Iowa, Iowa City, IA, United States; ^3^Iowa Cochlear Implant Clinical Research Center, Department of Otolaryngology, The University of Iowa Hospitals and Clinics, Iowa City, IA, United States

**Keywords:** cochlear implant, adults, music, problem solving, patient-centered research

## Abstract

**Background:**

Cochlear implants (CIs), which have been designed primarily to support spoken communication of persons with severe to profound hearing loss, are highly effective in supporting speech perception in quiet listening conditions. CI users as a group achieve significantly poorer perception and appraisal of music, and speech perception is compromised when background music is present, though outcomes vary considerably across recipients. A number of factors have been identified that contribute to variable music listening experiences, but many questions remain, particularly regarding experiences in everyday life from the perspective of CI users.

**Purpose:**

The purpose of this study was twofold: The first aim was to explore the perspectives of adult CI recipients regarding two experiences with music in everyday life: purposeful music listening and background music that competes with spoken conversation. The second aim was to develop a framework of everyday music experiences based upon CI perspectives that could inform future rehabilitative practices and research initiatives.

**Methods:**

Qualitative and patient-engaged research methodologies were used to emphasize the perspectives of the CI users. Participants included 40 experienced adult CI users ranging in age from 19 to 81 enrolled in 13 CI centers. Participants completed on-line semi-structured open-ended questionnaires regarding purposeful music listening and background music in conjunction with spoken communication. Responses were analyzed using an iterative inductive coding process consistent with grounded theory methodology. The interrelated themes that emerged from the data were then organized into a model synthesizing components from models on music response and self-management for persons with chronic health conditions.

**Outcomes:**

Data analyses informed the development of a Dynamic Problem Solving Model for Management of Music Listening Environments adapted from Hill-Briggs (2003) Problem Solving Model of Chronic Illness Self-Management. Key findings were: (1) Music listening is a dynamic, multifaceted experience; satisfactory listening depended upon optimal combinations of factors; (2) Music listening is effortful, but the extent of satisfaction is influenced by expectations and self-management of the situation; (3) CI users have limited access to resources for optimizing music experiences. Many CI users would consider rehabilitation, but level of commitment and priorities differ across CI users.

## Introduction

Cochlear implants (CIs) are designed primarily to support spoken communication and awareness of environmental sounds. Several decades of research and development to upgrade implant technology have resulted in enhanced speech perception, especially in quiet listening conditions, but many CI users still achieve poor outcomes for speech recognition in noise and for perception of music (Eskridge et al., 2012; Gfeller et al., 2012; Looi et al., 2012; Philips et al., 2012; Limb and Roy, 2014; Drennan et al., 2015; Dritsakis et al., 2017).

Most information regarding music perception and enjoyment by CI recipients has been generated through laboratory studies testing basic perceptual attributes (pitch, melody, timbre, rhythm) (e.g., Drennan et al., 2015 (for reviews, see Looi et al., 2012; Philips et al., 2012; Limb and Roy, 2014) or surveys using closed-ended (fixed response) questions deemed important by researchers (Gfeller et al., 2000; Mirza et al., 2003; Lassaletta et al., 2008; Migirov et al., 2009; Looi and She, 2010; Philips et al., 2012; Drennan et al., 2015). Nearly 30 decades of research indicates while some musical sounds are more accessible than others, CI users as a group have poor perception of pitch and timbre, and time spent listening to music typically declines following implantation (Gfeller and Lansing, 1991, 1992; Pijl, 1995; Fujita and Ito, 1999; Looi et al., 2012; Limb and Roy, 2014; Drennan et al., 2015). Interestingly, perceptual accuracy is not a strong predictor of music appreciation and CI users are variable in music perception and enjoyment (Gfeller et al., 2008; Philips et al., 2012; Wright and Uchanski, 2012; Drennan et al., 2015).

Factors contributing to variability include age, cognitive processing, residual hearing, hearing aid use, and music training (Gfeller et al., 2008, 2010, 2012). The impact of these factors varies as a function of the musical stimuli and response task (e.g., Gfeller et al., 2008, 2010; Fuller et al., 2018).

While accuracy does not typically improve from mere exposure over time (Gfeller et al., 2010), some aspects of perception and appraisal improve in response to focused listening practice or formal training, though outcomes are variable (for review, see Looi et al., 2012; Fuller et al., 2018). Despite potential benefits, music training is not commonly offered in typical clinical practice (Looi and She, 2010; Philips et al., 2012; Dritsakis et al., 2017).

Most studies of music and CIs have focused on purposeful listening. In everyday life, music also functions as background music for ambience or to enhance mood. Several studies indicate that background music has a negative impact on speech perception, but the impact varies depending upon the specific music (Eskridge et al., 2012; Gfeller et al., 2012; Başkent et al., 2014). Music and speech also interact through song lyrics (Collister and Huron, 2008). Hearing device users have particular difficulty understanding song lyrics if the SNR between sung lyrics and background accompaniment is too low (Gfeller et al., 2009; Wilhelm, 2016).

These studies have provided important information regarding basic aspects of music perception of CI users, with particular emphasis on device efficacy and assessment of “endpoint” outcomes in with controlled samples, stimuli, and environments (Pisoni et al., 2017). However, many questions remain concerning CI user variability in music experiences. Furthermore, laboratory testing cannot fully elucidate the challenges that CI recipients face when listening to music in everyday life (Philips et al., 2012; Pisoni et al., 2017).

Understanding the experiences that cochlear implant users face in everyday listening experiences with music is challenging in part because of the inherently complex and dynamic nature of music listening. Consider the following:

(1) Music in real life comprises rapidly changing and seemingly infinite combinations of collative properties of frequency, duration, timbre, and amplitude, resulting in ongoing changes in complexity and organized within different stylistic grammars. The listener’s perceptual organization, enjoyment, and symbolic meaning are affected by the extent of their familiarity with particular songs or genre, which is influenced by enculturation and training (Hargreaves and North, 2010). Furthermore, in everyday life, the functions of music vary (e.g., entertainment, part of cultural rituals, for relaxation etc.), as well as response tasks (casual listening, focused attention, etc.) (Sloboda, 2010; Hargreaves and North, 2010). In public settings, listeners often have limited control over which music is played and under what conditions (Sloboda, 2010; Hargreaves and North, 2010). Consequently, CI users are likely to be exposed to music with complex collative properties, including selections or styles that are unfamiliar.
(2) In real life, listening environments for music vary enormously (e.g., outdoor performances, concert halls, bars, personal listening devices, etc.), and the conditions change over time. For example, the number and configuration of other people in the environment, the sound waves within an acoustical space, and competing noise such as traffic or air handling systems will fluctuate over time.
(3) Music is commonly paired with speech through song lyrics or as background to spoken conversations in social settings (Gfeller et al., 2009, 2012); on-going changes occur in the focus and function of the music (Sloboda, 2010). In some instances, the listener attempts to understand sung lyrics against a background accompaniment (Gfeller et al., 2009; Wilhelm, 2016). Sometimes music is ambient background sound; the listener’s primary task is to extract the conversation (Gfeller et al., 2012). Music and conversation may co-occur in a task requiring split attention. For example, at a bar with live music, the listener’s attention shifts back and forth between listening to a jazz combo and the voices of their conversation partners.
(4) Listeners differ from one another and over time, not only as a function of auditory profile, but in psychosocial characteristics that impact listening (Hughes et al., 2018). People with hearing loss differ in self-efficacy and cognitive resources available for managing a complex listening environment or auditory signal (Smith and West, 2006; Gregory, 2011; Smith, 2014; Pisoni et al., 2017). Personal capacity for processing sounds will also change as a function of fatigue, age, or familiarity with the stimuli (Gfeller et al., 2008; Hargreaves and North, 2010; Başkent et al., 2014; Pisoni et al., 2017).

In summary, music experiences in everyday life are multifaceted and dynamic and thus challenging to represent in highly controlled experiments. To date, few studies with CI users have examined real-life situations involving music or addressed CI users’ perspectives and priorities for managing or improving music experiences (Looi and She, 2010; Plant et al., 2015).

While no singular research approach can fully address the complexities associated with real-life music experiences of CI recipients, some research methods are associated with *greater* emphasis on experiences as perceived by the target population. Two such approaches are patient-centered and qualitative research methodologies. Patient-centered research methodology seeks meaningful input from patients and other stakeholders (e.g., family members, advocates) throughout the research process (Clancy and Collins, 2010; Hickam et al., 2013; Brodt et al., 2015; Forsythe et al., 2017). Stakeholder input has been attributed with greater likelihood that the research questions and interpretation will reflect the perspectives and priorities of the target population, and that the methods will facilitate strong rates of enrollment, retention, and protocol compliance (Domecq et al., 2014; Frank et al., 2014; Sheridan et al., 2017).

The use of qualitative research in health care has risen markedly over the past 25 years (Bradley et al., 2007; Mather et al., 2018). Qualitative research can help reveal patients’ own experiences of illness and health care, as opposed to categories pre-determined by researchers, and can highlight previously unidentified issues worthy of future hypothesis testing. Qualitative research is particularly suitable for questions related to natural settings, such as coping with health problems in everyday life, thus complementing research questions better suited toward controlled experiments (Bradley et al., 2007; Creswell, 2014; Mather et al., 2018).

While there is no singularly appropriate way to conduct qualitative research, it has several hallmark characteristics. Questions are examined primarily through words rather than numbers. The data are reported in narratives, with liberal reporting of the participants’ own words; the researcher attempts to remain open to the participants’ perspectives (Savenye and Robinson, 1996; Bradley et al., 2007; Creswell, 2014). Qualitative research questions tend to be broad and exploratory in nature in order to encourage participants to share their experiences within the context of their daily lives and communities (Creswell, 2014). Rather than starting with a theory and an *a priori* hypothesis, qualitative researchers generate or inductively develop theory or pattern of meaning from the views of the participants.

The general process for analysis involves coding the data into categories and themes, and interpreting themes for relationships and core concepts. These are examined in relation to existing theories and literature. The researcher’s own experiences and background will shape their interpretation of finding, consequently, the authors reveal in the document prior experiences that may influence their interpretations; the use of first-person voice in describing the research process is common (Strauss and Corbin, 1994; Savenye and Robinson, 1996; Bradley et al., 2007; Creswell, 2014).

In-person or phone interviews, focus groups, and semi-structured questionnaires are often utilized in qualitative research to obtain rich narratives from participants (Savenye and Robinson, 1996; Creswell, 2014; Mather et al., 2018). However, in-person interviews or focus groups can raise challenges in gathering information from a geographically diverse sample. In addition, interviews can be challenging when conducting research with people with significant hearing loss. The propensity for misunderstandings due to competing noise, accents, or speech impediments, among other issues, can undermine clear communication and interact with the accuracy of their responses (Tates et al., 2009). Online questionnaires comprising open-ended items are reasonable alternatives for gathering interview data, particularly when seeking input from a geographically diverse population (Creswell, 2014).

Several recent studies have used qualitative methods to better understand the real-life experiences of cochlear implant users. Hughes et al. (2018) utilized a grounded theory analysis to explore listening effort in everyday spoken communication before and after implantation (Hearing aid use vs. CI use). Participants included 15 adult HA/CI users and two caregivers (ages 42–84). The participants responded to open-ended questions regarding listening effort. The conversations were transcribed, and units of meaning were coded and analyzed for themes. The analyses described the mental energy required to attend to and process spoken communication, and adaptation and compensatory strategies required to maintain control over their listening environment.

The analyses revealed that the CI improved the auditory signal enough to enable more successful communication. Using a CI moderated, but *did not remove* the requirements for listening effort. Listening effort, fatigue, and stress were problems with both HA and CI use, particularly in multi-speaker conversations. The degree of effort varied depending upon the level of background noise, information complexity, and speaker characteristics. CI users were more likely to “accept” the fatigue and effort required if the listening task helped them to maintain social connectedness. The authors reported that qualitative methods provided a more holistic and nuanced conceptualization of effortful listening in everyday life, and highlighted the importance of social connectedness as a motivation for sustained effort.

In some instances, qualitative research is used to better understand outcomes from prior experimental studies or standardized tests. Harris et al. (2016) used qualitative methods to better understand the highly variable patterns of rehabilitation strategies initiated by adult CI users in real life. A diverse group of 23 adult CI users completed open-ended questionnaire items and interviews; the data were analyzed using thematic content analysis. The analyses revealed that personal motivation and social support were critical to self-initiated rehabilitation, and emphasized the importance of the CI users’ attitudes and behaviors in optimizing CI benefit beyond basic device maintenance and mapping. Very few audiologists had offered information about or resources for rehabilitation.

Though not related specifically to music enjoyment, these studies (Harris et al., 2016; Hughes et al., 2018) present examples of qualitative research that focus on everyday listening experiences of CI recipients, emphasizing psychosocial factors and enhanced outcomes in real life. They also illustrate various methodological approaches suitable for examining everyday experiences and perspectives of CI recipients.

Focusing more specifically on the music experiences of CI users, Bartel et al. (2011) conducted qualitative case studies of 5 cochlear implant recipients from in their clinic who self-reported music as a major issue following cochlear implantation. Data were gathered through individual semi-structured interviews comprising questions about musical background, experiences with music prior to hearing loss (e.g., social life, profession etc.), and post-implant music appreciation. Grounded theory was used for data analyses. All participants considered music an important part of their lives prior to deafness; all experienced sub-optimal music appreciation after implantation—especially decreased sound quality and enjoyment. The five participants reported a wide range of satisfaction with music post-implantation and emphasized the need for careful listening. Participants expressed hope that rehabilitation might improve music experiences, but the study did not include questions about improving music listening.

Dritsakis et al.’s (2017) qualitative study also focused on music listening and CI users, but concentrated on the relationship between music and quality of life. The researchers enrolled 30 adult CI users between ages 18 and 81 representing a wide range of patient characteristics and musical background. As part of six focus group discussions, participants were asked broad, open-ended questions about music and quality of life. The transcribed conversations were analyzed using a deductive approach called template analysis. Pre-determined coding categories of physical, psychological, and social well-being based upon the Quality of Life Model of the World Healthcare Organization were used in the analyses.

Participants reported that music contributed to quality of life by influencing positive emotions, relaxation, reminiscence, and reduced isolation similar to experiences reported in research about persons with normal hearing. However, the participants also described unpleasant feelings and limited participation in music-related and routine activities because of difficulties with music perception and enjoyment. Background music with conversation required additional concentration and effort, and hindered social opportunities. The authors concluded that quality of life could be enhanced by improved music experiences, and recommended greater access to music rehabilitation. While this study emphasized positive and negative aspects of music listening and quality of life, strategies for, or barriers to optimizing music experiences were beyond the scope of the paper.

In summary, nearly 30 years of research indicates that adult CI users as a group have impaired perception and appraisal of music, though CI users are highly variable on many measures. Most research outcomes to date reflect endpoint outcomes in relation to device or user characteristics as examined in highly controlled studies.

These studies are essential to our understanding of CI benefit and the development of future technology. However, many questions remain regarding the real life music experiences of CI recipients, and those factors that contribute to positive vs. negative experiences with music. Few studies have focused on the psychosocial factors that influence music listening. Are there individual listener attitudes or behaviors that improve or undermine music listening, or that buffer disappointing outcomes? Do CI users have concerns that have been inadvertently overlooked in prior research? This study was initiated with hopes that the experiences and perspectives of CI users would offer fresh perspectives on those factors that support or undermine music experiences in everyday life.

The purpose of this study was twofold: The first aim was to explore the perspectives of adult CI recipients on two experiences with music in everyday life: purposeful music listening and background music that competes with spoken conversation in social settings. The second aim was to develop a conceptual framework based upon the perspectives of CI users that could inform rehabilitation practices and future research initiatives.

## Materials and Methods

### Approach

Because the purpose of this study was to better understand the perspective of adult cochlear implant users in everyday life, we chose two methodological approaches that would emphasize the viewpoints of the CI users themselves in naturalistic settings: patient-centered and qualitative research methods. Patient-centered research methodology seeks meaningful input from patients and other stakeholders in all phases of research (Hickam et al., 2013), which can result in findings that more fully reflect the perspectives and priorities of the target population (Domecq et al., 2014; Sheridan et al., 2017).

Stakeholder input was gathered in the initial development of our study, including one of the co-authors (A. Schwalje) who has a hearing loss, several CI users and family members affiliated with our center, and representatives of advocacy groups (State of Iowa Department of Disabilities, Association of Adult Musicians with Hearing Loss, University of Iowa Office for Student with Disabilities). Their input informed the development of research questions, questionnaire items, protocol, and data analyses.

Consistent with qualitative methodology, the research questions were broad, oriented toward the participants’ perspectives, and focused on naturalistic phenomena. Data were gathered through open-ended questionnaires, and data analysis comprised an iterative inductive process through which the data from the questionnaires revealed themes and core concepts.

In keeping with qualitative research methodology, we provide the context regarding the experiences of the authors that could influence their perspectives. The authors all had postgraduate training and professional experience as musicians. All three authors had professional experience testing or treating CI patients, and had engaged in on-going conversations with CI users regarding music and their CIs. The third author, who was a professional musician prior to becoming a physician, has a moderate progressive hearing loss and uses hearing aids.

Additional qualitative approaches associated with various portions of the inquiry will be described throughout the paper.

### Participants

Qualitative research utilizes purposive sampling in which participants are selected who represents particular phenomena relevant to the research questions (Savenye and Robinson, 1996; Creswell, 2014). Cochlear implant users comprise a diverse group with regard to auditory profile, life experiences, and musical experiences. Therefore, enrollment criteria were broad: CI users 19 years or older who have had at least 12 months CI use and who spoke English as a first language. While brand or model of device was initially taken into consideration, aside from the preservation of residual hearing, group data have not revealed particular devices or strategies as clearly superior for music perception (Gfeller et al., 2008; Looi et al., 2012). Thus, device type was not specified in enrollment criteria.

The University of Iowa Human Subjects Review Board approved enrollment procedures. Participants were identified through the Iowa Cochlear Implant Clinical Research Center’s CI Registry and through announcements posted on websites for CI recipients. Invitations including informed consent procedures were sent out to those who met criteria.

To ensure a diverse sample with regard to music perception and involvement, we examined the Iowa CI Registry database to ascertain distributions of pitch ranking tests (Pitch Discrimination Test, Gfeller et al., 2002) and a questionnaire of music background and involvement (Iowa Music Questionnaire, Gfeller et al., 2000). This perusal revealed a wide distribution of results on both measures, thus increasing the likelihood of enrolling a diverse sample with regard to music perception and enjoyment.

Invitations to CI users outside the center were posted on listservs and web postings including the following: Hearing Loss Association of the Americas (HLAA), the Association of Adult Musicians with Hearing Loss (AAMHL), or through word of mouth (e.g., informed by their audiologist). Those individuals interested contacted the center to participate. Each participant provided their consent in response to a letter of consent, and was then sent a link to the questionnaire.

Forty adults consented to participate, including 28 patients from the Iowa Cochlear Implant Research Center and 12 from other CI centers. This sample size was greater than enrollments recommended in textbooks about grounded theory analysis (20–30 being considered adequate, Creswell, 2014). The age range was 19–81 years at time of testing. Participants used the following devices: traditional long electrode devices (LE) (*n* = 22), hybrid (A + E) (*n* = 12), and single sided deafness (SSD, deaf ear stimulated by CI, normal hearing on the contralateral side) (*n* = 6). This provided a continuum of participants with regard to residual hearing, an important factor in music perception. The sample also included individuals with different onset of hearing loss and age at implantation. Twenty-eight had lost hearing well into adulthood; 12 had pre- or perilingual deafness and had reached adulthood by the time of this study. The average length of CI use was 12.25 years, ranging from 2.44 to 28.07 years.

While qualitative research does not conduct hypothesis testing of independent variables, themes that emerge from the data can reveal trends of subgroups within the population. Therefore, we examined different coding patterns from the subgroups (pre- vs. postlingual deafness, LE, hybrid, SSD) in later iterations the analyses.

### The Semi-Structured Questionnaire

Cochlear implant recipients’ perspectives were gathered through an on-line questionnaire. Open-ended questions were used to encourage participants to share rich and detailed thoughts, attitudes, and experiences, as opposed to responding to closed-ended (limited set) researcher-derived categories of response. A semi-structured approach, common in qualitative studies in health care, was used to encourage thorough responses closely related to the main research question (Creswell, 2014; Mather et al., 2018).

Initial questions for the online questionnaire were created by the authors. CI recipients, audiologists, and persons from advocacy groups examined drafts of the interview questions; their feedback was used to identify omissions and for input on recruitment, enrollment, and protocol implementation.

The questionnaire items reflected the primary research aims of the study, and included the following subtopics: (1) music enjoyment/experiences before hearing loss and after implantation; (2) speech and background music; (3) technology and music or music and speech; (4) music through CIs; (5) listening environments; (6) accommodations and strategies; (7) rehabilitation tools; (8) informational resources and counseling; and (9) aspirations for improved CI benefit with music. Closed-ended items were included to gather demographic information on the hearing profile, musical background, age, length of use, and type of implants used. The instructions and open-ended questions presented appear in Supplementary Appendix A.

### Protocol

An online questionnaire was chosen for several reasons: (1) It facilitated enrollment of individuals from multiple centers, thus representing a variety of clinical experiences; (2) Individual responded without influence of the opinions of others (which arguably can be advantageous, such as focus groups); (3) An on-line response mode reduced the possible impact of auditory deficits that might interfere with in-person group conversations; (4) Online response were downloaded directly into a database, thus avoiding possible errors in transcription or note taking. Prior research also indicates that outcomes from on-line inquiries are similar to or even superior to outcomes from in-person focus groups (Tates et al., 2009).

After informed consent was obtained, participants were contacted by e-mail with a password-protected link to a website using a Qualtrics^XM^ questionnaire platform. Participants were given 4 weeks to complete the on-line questionnaire. Each participant was paid $20 per hour for up to 2 h of time spent in completing their responses.

Each individual’s responses were automatically downloaded from the Qualtrics^XM^ platform into a protected database and stored using an alphanumeric ID. Each respondent’s information formed a unit of analysis. Questionnaire responses were copied to word documents and assigned a respondent code to prevent any bias toward familiarity with respondents or their device configuration.

### Analysis of Interview Data

**FIGURE 1:**
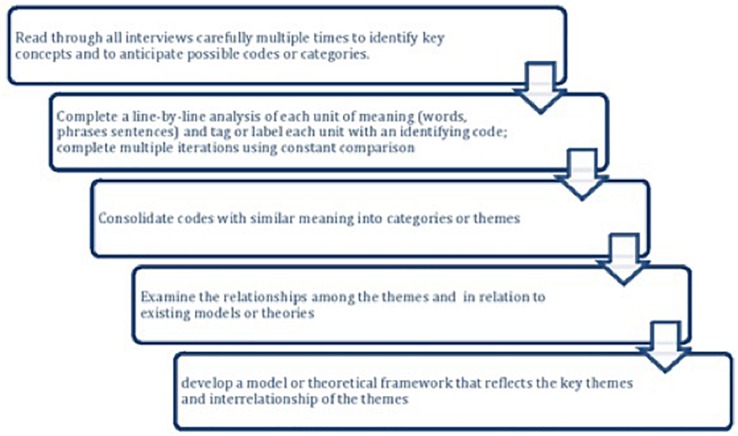
Flowchart of steps in the qualitative analysis.

The method for analysis was grounded theory (GT). The underlying premise of GT is that any potential theory is grounded in the coded data as opposed being based on *a priori* hypotheses. Consistent with GT, the analysis consisted of an iterative and reflexive process described below (see Figure 1).

The interview data were first read carefully multiple times by the authors to get a general sense of the data. In qualitative research, the text is dense and rich, and not all information can be included. Thus, researchers aggregate data into a small number of themes (Savenye and Robinson, 1996; Creswell, 2014). Proceeding line-by-line, the first two authors used open coding to break the data into meaningful units at the word, phrase, or sentence level. Each participant’s responses were analyzed independent of the individual questions because responses may apply to multiple questions.

Each unit of meaning was assigned a conceptual label or code to define meaning, actions, and to facilitate exploration of relationships between codes. Codes may include expected concepts from past literature as well as codes not anticipated (Savenye and Robinson, 1996; Bradley et al., 2007; Creswell, 2014). Responses that fit under multiple codes were included under each relevant code.

The first two authors completed a second round of coding (focused coding) to group similar concepts; those codes were once again grouped into more abstract, high level categories which are referred to as themes (Bradley et al., 2007). For example, codes of pitch, complexity, and timbre were grouped under a higher-level category of “structural components of music.” Codes such as “structural components of music” and “environment” could be grouped under themes such as “pleasant” or “unpleasant.” In some instances, a code fit under more than one theme. For example, the code, “loudness” was not only a structural feature of music but also related to codes of unpleasant experiences. During the first two rounds of coding, the readers were blinded to participant identity or device subgroup.

Although qualitative methods focus primarily on the words, magnitude coding can help determine the most prominent and important themes (Saldaña, 2013). Frequency (i.e., number of occurrences) and extensiveness (number of respondents) of responses were calculated (Attride-Stirling, 2001; Miles et al., 2014) (see Supplementary Appendix B). Following the first two rounds of coding, the first two authors examined the data in relation to device subgroups (LE, Hybrid, SSD) for possible trends related to hearing configuration. The most prominent themes and the relationships among themes were conceptualized through core categories or central concepts, which represented the main themes (Creswell, 2014; Hughes et al., 2018).

Consistent with GT methodology, further literature review followed data analysis to support and further develop the theoretical categories originating from the data (Savenye and Robinson, 1996; Creswell, 2014; Hughes et al., 2018). This included models or theories from music psychology, audiology, and health psychology (e.g., self-management of chronic illness).

Verification of coding was established through ongoing discussion between the first two authors regarding the quality and consistency of coding. In addition, an outside reviewer (a research assistant from the lab not involved in this project) analyzed a 20% subset of responses to verify the coding used. Responses from the outside evaluator were highly consistent with the initial analysis; no categories or codes were recommended for addition or deletion and any differences in coding choices were resolved through discussion.

Validation of content was achieved through member checking (Creswell, 2014). This involved sharing a summary of themes from the analysis with the participants, to determine whether the themes reflected their comments. Thirty of the 40 participants responded to the invitation to participate in member checking, and confirmed the themes to be representative of their questionnaire responses.

Consistent with grounded theory methods, the results were reported in narrative description of the primary themes or concepts and dissenting opinions, with liberal use of direct quotes from the participants as exemplars (Creswell, 2014).

## Results

### Codes and Themes

Across the 40 participants, the 27 open-ended questionnaire items yielded a total of 601 individual responses comprising 1655 lines of typed text, numbering 21,520 words. Supplementary Appendix B presents the coding definitions as well as the themes, codes, and sub-codes that resulted from three rounds of inductive coding. This appendix presents the number of and percent of participants represented in each code. While qualitative data relies primarily on narratives to report the results, magnitude coding can assist in identifying the most prominent themes or codes. By examining the prevalence of and relationships among codes and themes, we identified three core categories. Core categories are central concepts that appear frequently, represent main themes, and are related to one another (Creswell, 2014; Hughes et al., 2018). These core categories appear below.

### Core Categories

#### Core Category 1: Music Listening Is a Dynamic, Multifaceted Experience, With Satisfactory Listening Conditional to Optimal Combinations of Factors

Music experiences in everyday life involved dynamic interaction of the CI recipient’s auditory profile, the auditory signal (music, music and speech), social context, and the environment. These components changed over time, and were often beyond the control of the listener.

**FIGURE 2:**
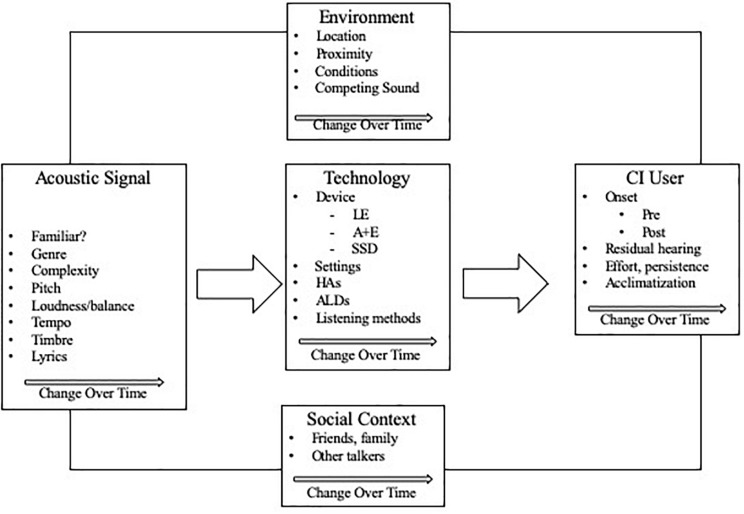
Codes and themes associated with core category 1.

Particular factors (e.g., music, environment, and social context) were not inherently positive or negative, but rather conditional to prevailing circumstances over time as they interacted with the listener’s auditory profile. Qualifiers such as “it depends upon,” “it varies,” “sometimes,” “it differs when/if” were commonly found in relation to both positive and negative experiences. For example, under the code of “environment,” one participant described a sub-code of “outdoor concerts” as pleasant and conducive to enjoyment. Contextual details revealed that this outdoor concert was held in an open, quiet park and the CI user was seated in optimal proximity to the performers. In contrast, another participant described an outdoor concert as a poor listening environment, noting that the concert venue was in close proximity to noisy traffic, and the social context of the concert was a noisy audience. Codes and themes from the data related to this core category appear in Figure 2.

#### Core Category 2: Music Listening Was Effortful, but the Extent of Satisfaction Was Influenced by Expectations and Self-Management of the Situation

Most participants described music listening as effortful or requiring attention; fatigue, cognitive overload, and emotional frustrations were associated with negative experiences or avoidance behaviors. While many listening conditions were beyond the control of the CI user, participants varied in their response to and success in handling challenging conditions. Experiences in managing music listening comprised several themes, including strategies used to engineer the listening situation (e.g., music, environment, technology), barriers that impeded enjoyment (e.g., environment, lack of resources), and personal characteristics (e.g., attitudes, perseverance, avoidance) that influenced the listeners’ responses to evolving circumstances, and attitudes toward those experiences.

#### Core Category 3: CI Users Have Limited Access to Resources for Optimizing Music Experiences

Most participants had limited awareness of, or access to training programs, assistive listening devices, or other resources for enhancing music experiences. The overwhelming majority (90%) expressed a desire for more resources to improve music experiences. However, participants described excessive cost, lack of access, and time required for training as barriers to rehabilitation. Level of commitment (e.g., willingness to dedicate time and money) and priorities for training content varied across participants.

Together, these core categories indicated that satisfactory outcomes in everyday music experiences were not only a function of the musical sounds, the auditory profile of the listener, and the listening environment, but also attitudes and behaviors required for managing or coping with the degraded signal, especially in inhospitable listening environments. Unfortunately, resources to help CI users improve listening enjoyment were not readily available to many of these participants.

In order to address the second aim of this study, we developed a conceptual framework, based upon the CI users’ perspectives that could inform future research and rehabilitation.

### Generating a Conceptual Framework for Rehabilitative and Research Options

Consistent with grounded theory, we conducted an additional literature review after the initial coding process to examine themes, categories and their interrelationships as they related to existing models and theories. A model from psychology of music, the Reciprocal Feedback Model of Musical Response (RFM-MR) (Hargreaves and North, 2010), conceptualized the reciprocal nature of music, the listening situation, and the listener, resulting in physical, cognitive, and emotional responses to music. The RFM-MR reflected many codes and themes associated with our first core category, but did not address the perceptual limitations imposed by hearing loss or the CI, which severely undermines perception and enjoyment of the collative characteristics of music. The RFM-MR also did not address strategies or resources required to manage difficult listening conditions.

**FIGURE 3:**
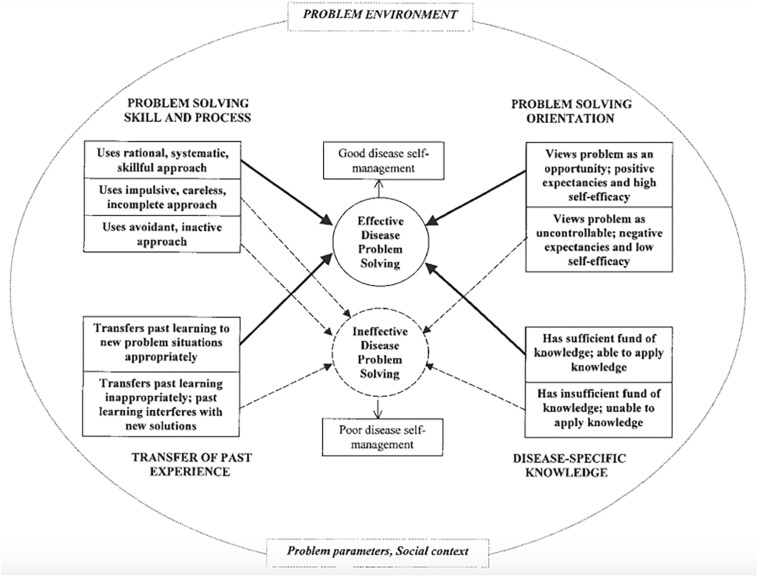
Hill-Briggs (2003) problem solving model of chronic illness self-management.

Until medical and technological solutions can more nearly restore “normal” musical sounds, CI recipients seeking more satisfying music listening are left to manage their circumstances by establishing realistic expectations and rehabilitative and compensatory efforts. These can be facilitated by the internal (cognitive and emotional) state of the listener (Krueger and Casey, 2009). The Hill-Briggs Problem Solving Model of Chronic Illness Self-Management reflected many codes and themes associated with core categories 2 and 3 (see Figure 3).

Hill-Briggs developed her model to guide disease self-management training and interventions for diabetics, drawing extensively from cognitive psychology, educational research/learning theory, and social problem solving. While diabetes is clearly different from hearing loss in etiology and treatment, both diabetes and hearing loss are chronic conditions that require symptom management in the absence of a cure. Psychological and behavioral factors play a critical role for both populations (Hill-Briggs, 2003; Harris et al., 2016; Young-Hyman et al., 2016; Hughes et al., 2018).

Diabetes can be managed to differing extents by diet, exercise and medication, but compliance with protocols and complete control can be challenging; even individuals who are highly compliant experience medical complications (e.g., loss of vision, nerve pain) and frustration (Hill-Briggs, 2003; Young-Hyman et al., 2016). Furthermore, dietary options associated with social environments and the desire to “fit in” can undermine compliance with nutritional guidelines and exercise. Personal factors such as fatigue or motivation also impact successful management.

Cochlear implants provide access to sound, but they do not cure hearing loss or replicate normal hearing. The CI user is responsible for device maintenance and use, compensating for poor auditory input, and negotiating the many noisy environments found in everyday life. CI users may face choices of “smiling and nodding” in noisy and tiring listening conditions, or avoiding those circumstances altogether; avoidance contributes to social isolation, which has implications for quality of life and emotional well-being (Philips et al., 2012; Hughes et al., 2018). Environments with music are particularly problematic because CI technology is not well suited to conveying key structural components of music (for review, see Limb and Roy, 2014). These challenges described for diabetics and CI users respectively, influence the extent to which the individuals, even with cutting edge medical interventions, will thrive and enjoy satisfactory social integration.

#### Model Development

**FIGURE 4:**
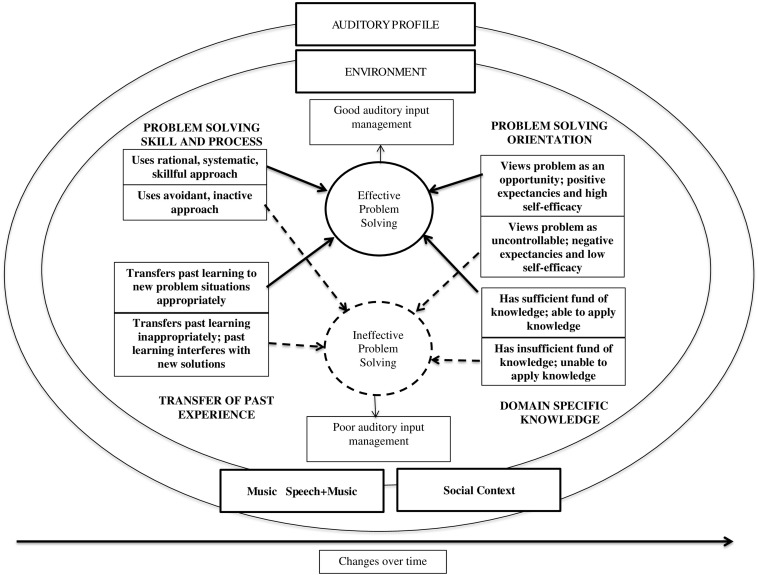
Dynamic problem solving model for management of music listening environments adapted from Hill-Briggs (2003).

While the RFM-MR (Hargreaves and North, 2010) focused on music, the listening situation, and the listener, the Hill-Briggs (2003) model focused on cognitive, affective, and behavioral responses to a chronic health condition within a social environment. Examining the emerging themes and core concepts from our data analysis in relation to these two models, we synthesized the relevant components of the two models to conceptualizes the CI users’ perception of music in everyday life in a model that we call A Dynamic Problem Solving Model for Management of Music Listening Environments (DPSM-MMLE) (see Figure 4).

The outer elliptical components of the model (auditory profile, environment, music and music and speech, and social context) reflect the reciprocal process as conceptualized in the RFM-MR (Hargreaves and North, 2010). The broad category of “Problem Parameters” from the Hill-Briggs model was changed to “Music and Music and Speech,” the focus of the research question. Given the diversity among CI users (e.g., age of onset, residual hearing) in relation to music listening, we included a component of “Auditory Profile.”

The listener’s responses to or handling of the music experiences appear within the ellipses: cognitive, affective and behavioral processes associated with self-efficacy, problem solving and self-management associated with the Hill-Briggs model (Hill-Briggs, 2003). In the adapted model, the category of “Disease Specific Knowledge” from Hill-Briggs’s model was modified to reflect knowledge of hearing loss and CI technology in relation to music (“Domain Specific Knowledge”). Finally, to emphasize the dynamic and reciprocal nature of music, listening environments, and listener attributes and actions (e.g., neuroplasticity, changing attitudes and behaviors) we added the component of “Changes Over Time.”

Consistent with qualitative methods, the following section presents narratives and exemplar quotes describing the core categories, themes and codes as conceptualized within the Dynamic Problem Solving Model (DPSM-MMLE) for Management of Music Listening Environments.

#### Core Categories and Themes Conceptualized Within the Dynamic Problem Solving Model for Management of Music Listening Environments

##### Core Category 1: Music listening is a dynamic, multifaceted experience, with satisfactory listening conditional to optimal combinations of factors

Four components of the DPSM-MMLE (outer ellipses) reflecting Core Category 1 were: (1) music and music in speech, (2) environment, (3) auditory profile, and (4) social context. As noted in the introduction, each of these components is dynamic—that is, characterized by change. Furthermore, music listening is multifaceted in that all of these components interact. Music heard in everyday life (as opposed to controlled stimuli in experiments) is inherently dynamic and multifaceted in that it can include from one to as many as hundreds of individual musicians (such as in a large symphony or choral performance) performing together on rapidly changing combinations of pitch, rhythm, timbre and dynamics; these structural elements will continue to change second by second in overall complexity and familiarity to the listener. These musical combinations interact more or less successfully with the processing characteristics of the hearing device(s), and are impacted by the changing room acoustics, concurrent social interactions, and the presenting cognitive processing capabilities of the listener.

As documented in prior studies, CI users typically have impaired perception of some musical features, particularly pitch, melody, and timbre, even when presented as highly controlled individual components. By in large, loud environments are uncomfortable or distorted. These participants responses, while consistent with those findings, offered a more nuanced picture specific to everyday experiences, namely that there is a continuum of positive to negative experience for each of these factors (environment, musical features, auditory profile, social context) contingent upon the specific combination of elements at any given point in time. These factors were to a considerable extent beyond the control of the CI user.

The conditional and multifaceted nature of music listening was illustrated in the following quotes:

“Experience with music and with the CI varies greatly with factors such as the quality of the speakers and whether I’m familiar with the music. If I’m listening to a song I’m familiar with on Bose speakers in a quiet small room, it is a great experience. If I’m listening to an unfamiliar song on a cell phone, it is an awful experience that requires a lot of effort” (Postlingual LE).

This participant described not only the impact of elements of the music impact but also the importance of manner of listening and the location. Another traditional implant recipient and a hybrid user elaborated on the impact the sound environment can play.

“I feel like the difficulty of speech and music increases a lot with increasing environmental factors, such as the size of the room/area, or the number of people speaking, or the number of instruments involved. It requires a great deal of effort to enjoy music or understand speech toward the high end of the spectrum of unfavorable environmental factors” (Postlingual LE).

“It depends on the situation. At the symphony, it is easy to enjoy. In a restaurant or department store, it is difficult to understand the spoken word when music is playing. At a social gathering, music makes it difficult to understand conversation. Music is easy to listen to by itself but mix in conversation and it is difficult to hear either conversation or music” (Hybrid, Postlingual).

This quote illustrates the dynamic interaction of specific musical elements, the CI, and the listener’s present energy status. “There are some tones that are clear as a bell and some tones with static-like noise to them. Some days seem better than others. Maybe I get tired?” (Hybrid, Postlingual).

Specific to music characteristics, participants emphasized particular features of music that influenced music listening. The most prominent codes were: (1) prior familiarity with the music “I do not understand any of the words during songs unless I already know the song;” (2) complexity of the music; and (3) loudness: “Fast paced and loud is difficult, where soft and slower are more easily understood. Men’s voices are easier to understand than a women’s high voice.” (LE, Prelingual). Many found familiar music easier to understand, and “new” music difficult or impossible to understand. Participants used mental recall of familiar music along with most accessible structural components to piece together the degraded signal.

“I was in a toy store and over the speakers they were playing The Mickey Mouse Song. I used to watch the Mickey Mouse Club when I was 5–7 years old and just loved the show. At first I ignored the music but then I recognized the beat and suddenly I realized what the song was. I could hear the lyrics and the memory of that song came flooding back to me. I stood there with my mouth open. That was an awesome moment.”

Regarding complexity, 67.5% of participants indicated that music with fewer instruments or voices, or made up of simpler melodies, harmonies or rhythms were easier to perceive and organize. Participants reported, “If there are a lot of instruments playing together, it just sounds like noise” and “The more complex the musical harmonies and music types, the worse it sounded”.

Some musical genres were associated with more accessible components. For example, one participant stated, “Country music is best to understand because the background music isn’t so loud. You can hear the words better. If I try rap or rock it is hard. I would say it depends more on the song (the level of background music playing)” (LE, Prelingual).

Eighty-five percent of participants described one or more structural features of music as barriers to enjoyment. Nearly half (47.5%) described loud music as a barrier to satisfactory listening, whether as part of purposeful music listening or in combination with speech. Poor balance among components within one piece of music, such as an accompaniment too loud for the vocalist, or melody line was problematic. Only 17.5% specifically mentioned pitch (frequency range, matching a pitch, or recognizing melodies) as a barrier to music enjoyment.

With regard to speech and background music, background music was described in negative terms, particularly when loud in relation to the target conversation partner(s).

“Depending upon the situation [background] music doesn’t have an impact on my conversations with people because it’s soft and calming. However, at a bar…it’s harder to hear in a social conversational setting because … they generally have it turned up louder than they need to have it” (LE, Prelingual).

The impact of loud music was repeated across all participants: “Depending on how loud the music is, I do have challenges understanding people in group settings.” “It is very difficult to understand and carry on a conversation when music is playing at a restaurant or social gathering.”

###### Environment

Ninety percent of the participants described the environment as a barrier to satisfactory listening. For example, sitting too close to sound speakers could make the sound intolerably loud, or drown out the voice of one’s conversation partner. In contrast, being seated too far from a performer could make perception problematic. Problem environments were considered a barrier across all subcategories of CI users (onset, device type).

“Location of the music makes a big difference in music enjoyment. I have purposefully avoided going to live concerts. If I try to listen to music and there are other conflicting sounds such as machines running or people speaking in the background, then it becomes a waste of time. For me, the best environment would be listening to music on a car radio with the car parked in a parking lot and the windows rolled up with the motor turned off. That’s zero interference and I can control the volume and sound on the car radio” (Postlingual LE).

###### Social context

Music experiences often occur in social circumstances as entertainment, cultural enrichment, or as background ambience (Sloboda, 2010). Thus, social context is an important aspect of music experiences, and can be either negative or positive in nature. The participants commonly described social events as negative experiences due to multiple talkers. Although ambient music in social settings is typically intended to create a pleasant social environment, ambient music can function as a masker to the speech of conversation partners. “It is very difficult to understand and carry on a conversation when music is playing at a restaurant or social environment” (Postlingual LE).

Many participants expressed frustration regarding situations requiring focused attention to both speech *and* music (such as live music at a club), which resulted in unsatisfactory outcomes for both. Participants described undesirable options of either putting up with loud sounds and having to “nod and guess,” or avoiding many social situations altogether. Social settings combining music and speech tended to be problematic for all subgroups of the participants, regardless of the differences in residual hearing (LE, SSD, Hybrids).

“Awful! The problem is that virtually all good restaurants are noisy.” This was the response from one postlingually deaf LE participant. Another stated, “Bars can be awful. Either too loud to hear people talking or all the people talking makes it so I can’t clearly understand the music” (post LE). When trying to participate in situations where background music was present, fatigue was a factor: “I often get exhausted from trying to hear and will just zone out.” (Post LE) “Oh boy, carrying on a conversation in a noisy setting is difficult work.” (Hybrid LE)

Participants also described the problems associated with background music in the context of film scores for movies or TV: “I can do one or the other… I have never been able to listen to two things at the same time… For TV, I still rely heavily on the CC [closed captioning].”

While the noise that accompanies many social gatherings was problematic, other comments revealed positive aspects of social context. Approximately a quarter of the participants (27.5%) indicated that social support from others in one’s group helped CI users to cope with difficult listening situations. As one participant with single sided deafness stated, “I’ve trained my friends to sit on my good ear side so I can hear them talk and also hear music.” Another participant shared, “If I’m in a social situation at someone’s home, I may ask them to turn off the music as it interferes with my ability to understand speech” (Postlingual LE).

More than a third (37.5%) of the participants discussed the benefits of information shared among CI users, such as information on assistive listening devices (ALDs), strategies, and resources. A hybrid user reported, “I have 3 uncles who got the cochlear implant prior to myself, and I spent several hours discussing the ins and outs of the device with them, therefore they have been my primary resource.” Another participant with pre-lingual deafness stated, “It was always interesting in HLAA groups to hear about other deaf/hard of hearing individuals’ experiences in complex listening environments and what they did or did not do to fix the problem.”

###### Auditory profile

Trends across the subgroups (LE-Pre or postlingual, Hybrid, SSD) indicated that the listener’s auditory profile and hearing devices had an important impact on music listening, depending upon the particular musical features presented. However, participants within all device configuration groups described music listening as effortful, particularly if the music components were complex or the listening environment was noisy. Postlingually deaf individuals often mentioned the use of top-down processing to make sense of degraded input, while prelingually deaf individuals tended to describe music as more enjoyable than the other subgroups; they seemed more satisfied with what they could access through their CIs.

###### Residual hearing

Persons with SSD and hybrid devices had greater residual hearing, which had a synergistic impact on electric hearing. While very few comments included specific reference to pitch, melody, harmony, or timbre, persons with more residual hearing (e.g., SSD or Hybrid users) tended to find greater enjoyment in pitch-based components of music.

###### Device configuration

Most differences in perceptual accuracy or enjoyment were associated with residual hearing or onset of hearing loss, rather than technical choices. Very few participants offered comments regarding the CI brand or signal processing scheme, though a few did report device-related enhancements, such as this prelingually deaf LE user: “I could not imagine life without music. Since getting the Nucleus 6, I notice an improvement in hearing the word/lyrics in the songs such as country music. Very enjoyable.”

More common were general comments regarding the limitations of CI, signal processing, or ALDs. “I have found that programming for music is lacking (Postlingual LE). “The quality I have now [with my CI] does not make music sound rich and full.” (Postlingual LE) “I have tried to have a music program placed onto my processors. To be honest, the software used to program these must be very bad” (Postlingual LE). “I have never liked any of the automatic programs… I have tried these devices with CD players and get either a buzz, too soft as well as distortion” (Postlingual LE).

Participants described Roger Pens (small wireless microphones that looks like a pen) and other mini mics most frequently as helpful ALDs. A small proportion used hearing aids in conjunction with their CIs; those using hearing aids generally described them as helpful. However, described enhancements of technology were often conditional to specific kinds of music or listening situations.

###### Changes over time

As prior research indicates, music perception and enjoyment does not normalize as a result of mere exposure and on-going CI use, however, extended and focused listening time can enhance music listening (Gfeller et al., 2010). As noted earlier, music listening experiences are inherently dynamic, with musical combinations, environmental factors, and the listener’s internal state changing over time. Related to this concept, one participant noted that she could function well at the ceremonial part of a wedding, but as soon as the reception begin, the loud music made coping impossible and she was “forced to leave.” In addition, changes over time related to the listener’s internal capacities. The participants described changes in energy, cognitive or emotional resources, or acclimatization to the device or signal processing that influenced music enjoyment.

One participant described changes in acclimatization to the signal over the course of time:

“The first 3 months after activation was extremely difficult. As time went on, it became easier. About 9 months it started sounding “normal” again. I was still missing things but my brain adjusted to the new sound and it was becoming enjoyable again rather than effortful. Now at 1.5 year, it is easy and enjoyable probably 90% of the time” (Hybrid, Postlingual).

Another listener, with a traditional device also shared their sentiment.

“For 3 years I struggled just to get decent sound in this ear [second implant]. But after more and more programming, it slowly improved (not as good as the first [CI] through)…At first, I couldn’t tell if I got the correct notes [while playing the piano]…but interestingly I found that once my fingers remembered the fingering, the scales sounded right” (Postlingual, LE).

Looking across themes and codes associated with Core Category 1, a major theme that emerged is that music listening was effortful. As one participant stated, “After implantation, I needed to totally relearn music.” This theme emphasizes that technology alone did not facilitate meaningful listening experiences with music; attitudinal and behavioral characteristics of and strategies used by the CI recipient were as important. That is the focus of the following section.

##### Core Category 2: Music listening was effortful, but extent of satisfaction was influenced by expectations and self-management of the situation

**FIGURE 5:**
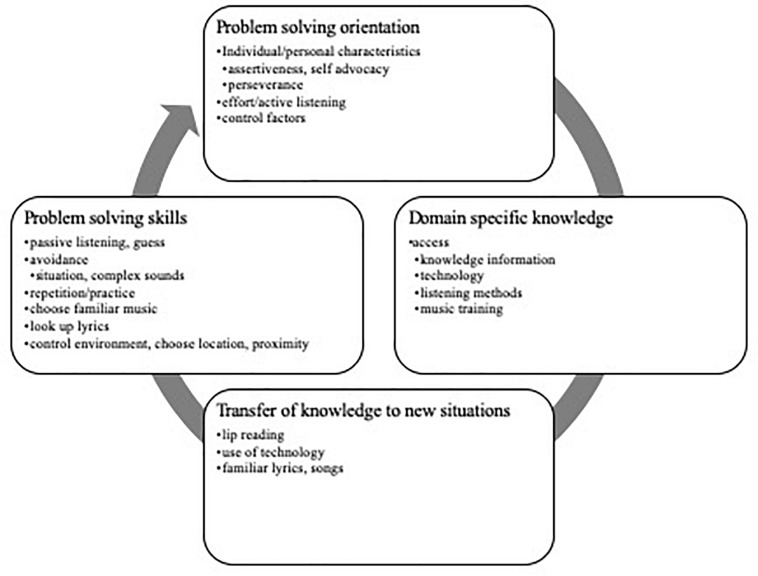
Codes and themes associated with core category 2.

Most participants described music listening as effortful. However, the extent of satisfaction in listening could be mediated by the internal capacity of the CI user to cope successfully or manage the music, environment, social context, or use of ALDs. Considering relationships of strategies (uses of technology, musical choices, social support, and attitude) and the barriers that undermined satisfactory listening, these themes were examined in relation to self-management approaches identified in health psychology (Hill-Briggs, 2003). The following section summarizes themes and codes that reflect the following quadrants within the ellipses of the DPSM-MMLE: domain specific knowledge; transfer of past experience to new situations; problem solving skills, and problem solving orientation. The themes and codes that emerged from the data appear in Figure 5.

###### Domain-specific knowledge

Self-management of challenging circumstances required adequate knowledge of hearing loss and hearing devices as they interacted with music components, as well as resources for optimizing music (e.g., training programs, accommodations). These participants possessed the greatest amount of domain specific knowledge about CIs, hearing aids, lip reading, closed captioning, and ALDs. While 89.47% indicated that they would like to have access to music training, most were unfamiliar with options or unsuccessful in acquiring resources. Problems with access are discussed in greater depth later in this paper (Core Category 3).

###### Transfer of past learning

The ability to recall and apply past knowledge appropriately to new situations is an important aspect of problem solving (Hill-Briggs, 2003), though transfer is predicated on sufficient knowledge of relevant information. Participants described examples of transfer of knowledge related to hearing loss (e.g., lip reading, closed captioning) as well as music. In some cases, past information was not fully relevant (Hill-Briggs, 2003). For example, some applications of ALDS were unsuccessful, particularly in relation to music listening.

Many relied on closed captioning for TV or movies. A number (32.5%) of participants used lip-reading not only to understand conversation partners in noisy environments, but also to understand song lyrics. For one participant, lip reading paired with technology was useful in live concerts.

“I enjoy going to concerts, but in order for me to follow along with the lyrics, I have to look at the big screen to see their lips. Overall I enjoy my concert experiences and I will continue to go to more because I enjoy country music because that’s the type of music I enjoy best” (LE post).

Knowledge of specific music also helped: “Having as much information as possible about the music being played is a trick of mine. I’ll look at the set list ahead of time to know what is coming next” (LE Prelingual).

###### Problem solving skills and process

The data revealed important differences among the participants with regard to problem solving skills and processes (e.g., avoidance vs. systematic, skillful approaches). Active strategies used for management or coping included: (1) using technological options such as Roger Pens, closed captioning, adjusting the volume/sensitivity, (2) personal effort, including lip reading, assertiveness, effortful/active listening, repeated listening, (3) careful music selection (familiar music, or more accessible genres), and (4) controlling the listening environment (proximity, listening conditions). However, these strategies required initiative, trial and error, effort and a personal orientation toward problem solving, which is discussed later in this paper.

The effectiveness of particular problem solving strategies varied as a function of the hearing history, device characteristics, musical background, and willingness to seek social support. For example, comments regarding using one’s memory of music to “piece together” previously familiar music were associated with postlingually deaf participants. “Where there is background music, I ask the people I am with what song is playing and then pull from auditory memory” (LE pre).

Many of the participants used avoidance of problem situations (52.5%) or the sound source (37.5%) as a passive strategy for coping. It was highlighted by these participants, “I don’t go out very much because it’s very difficult to hear in social settings” (Prelingual LE). “I have purposely avoided going to live concerts because I know I would not enjoy it.” Another hybrid recipient reported, “Social situations with music (wedding receptions)—I leave.” In regards to movie theaters, one prelingual participant shared, “I purposely do not go to the movie theater because I know I would not enjoy it” (LE, Prelingual).

Possessing relevant knowledge does not automatically result in effective transfer or knowledge or effective problem solving (Hill-Briggs, 2003). More positive attitudes toward challenges have been described as problem solving orientation.

###### Problem solving orientation

Coding revealed that CI users differed considerably with regard to their problem solving orientation. Problem solving orientation in this context referred to a strong sense of self-efficacy, viewing problems as opportunities, and positive expectancies. Comments from 42.5% of the group reflected some level of self-efficacy, that is, confidence in the ability to handle a situation; those with strong self-efficacy took initiative to improve listening circumstances. For example, one CI recipient stated,

“I feel it is up to the CI user to step up and ask what the conversation is about. and make them [conversation partners] aware of my presence. Training should include straightforward discussion with CI users that they need to take control in their conversational groups” (LE post).

Another added, “I believe, in order for the cochlear implant to work, you have to be an active participant in working/training yourself to listen with it.” A third, highlighting the need for self-advocacy shared, “I’ve been known to ask the hosts to turn [loud music] down/off, and it usually ends up that other people thought it was too loud as well but didn’t want to say anything.” (Postlingual LE) This sentiment was echoed by another, “If I’m going to be in a restaurant, I will often ask for a corner or a quiet table. Sometimes I will ask to have the music lowered (Postlingual LE).

Another CI recipient implanted as a child stated, “I usually ask if we can go to a more quiet place, or I try my best to read lips.”

In some instances, problem solving orientation involved positive expectancies more than strategies or accommodations. “I go to a lot of rock concerts and while I may not have complete quality sound that I can enjoy without lots of effort, I have not made it a factor when determining whether to go to a show or not” (perilingual LE).

In contrast, the following quotes, just a few of many, illustrate a lack of control or low self-efficacy: “Sometimes I’m just lost and can’t wait to go home” (Postlingual hybrid recipient). “I do avoid noisy environments and sometimes avoid a situation altogether” (Postlingual LE).

##### Core Category 3: CI users have limited access to resources for optimizing music experiences

This core category related to the component of Domain Specific Knowledge, one of the four central quadrants of the DPSM-MMLE. A key concern of these participants (82.5%) was a lack of accessible and/or affordable resources for dealing with everyday listening experiences involving music. Sixty-five percent of the participants indicated that they were unaware of resources or information to enhance CI benefit, and that rehabilitation was not addressed in regular audiology appointments. As one participant noted, “There is often a gap between the medical community, including doctors, audiologists and manufacturers of hearing devices and the real world of living with a hearing loss.” Another stated, “What would help? Audiologists who believe that listening to music with CIs is possible.”

Ninety percent of participants indicated that they would like to improve music listening. Of that group, 72.2%, especially LE and hybrid users, indicated that they would be interested in trying music training, especially computer-based training. These themes are illustrated by the following comments:

“I would suggest that there be more emphasis placed on the emotional adjustment to living with a CI. I received my implant in December of 2016 and is just now feeling settled. It was a tumultuous first year. I worked very hard to remain positive” (Post hybrid).

“I think that everyone who is implanted and then with every change of the implants (hybrid to full electronic) should be able to go to rehab much like those who have problems with their physical body. If you were able to go weekly and someone was able to explain the features of the implant over and over and could help you track your progress that would be helpful!” (Post hybrid).

This desire was echoed: “I would like to see a speech/hearing rehabilitation/therapy program that CI users could access in person with trained professionals to become proficient at using every possible technology available for the CI’s to improve their overall listening experience” (Post hybrid).

Many participants expressed uncertainty on where to find resources, noting limited counseling time with audiologists, and lack of non-commercial websites that include information about music, especially for adults. Over half the participants (57.5%) indicated that cost would be a barrier to training or purchasing ALDs that might enhance music listening. The participants described an “acceptable” range of cost for training programs as “free” to less than $100. Nearly three quarters (73.68%) indicated that they would prefer computer-based training that they could complete from home at their own convenience, though some desired social support (e.g., on-line feedback) as part of that training.

“I would be more comfortable doing it online if it is self-paced and measures my progress. It would be helpful if I could communicate with a person to ask questions and maybe share my progress and experience with other CI users.”

Nearly half (47.3%) remarked that time required for training, even if affordable, would be difficult to accomplish along with normal life responsibilities. Those expressing interest in training indicated that they would be interested in training for a few half hour or hour sessions, several time a week for a few weeks duration.

With regard to content, some expressed concern that available training programs lack relevance to their real-life needs. The top priorities (>25%) for improvement were: understanding lyrics (63.16%), enjoying personally favorite genre (55.25%), improved listening of more complex music (31.58%), and more normal sound quality (28.9%).

## Discussion

The first aim of this study was to explore the perspectives of adult CI recipients regarding music in everyday life, in essence, asking them to “think outside the booth.” Core categories that emerged from the data included: (1) the dynamic, multi-faceted, and “conditional” nature of music listening; (2) problem solving attitudes and behaviors that support enhancement or coping; and (3) the limited resources currently available for helping CI users to optimize music experiences in real life. The components associated with these core categories were organized into a framework, the Dynamic Problem Solving Model for Management of Music Listening Environments.

The results of this study confirmed that many prior music-CI studies address priorities of CI users, as well as revealing concern that some CI recipient priorities have heretofore have received limited research attention. Consistent with prior research studies, these participants reported difficulties perceiving and enjoying many aspects of music and understanding speech in background music. The range of responses was also consistent with variability documented among CI recipients for music outcomes (Gfeller et al., 2008, 2010, 2012; Looi et al., 2012; Başkent et al., 2014; Limb and Roy, 2014; Drennan et al., 2015).

Not all their priorities were in line with research trends to date, however. For example, present-day research tends to emphasis “endpoint” results specific to device or processing categories (Philips et al., 2012; Pisoni et al., 2017); these participants offered very few comments regarding specific brands or models of CIs or signal processing. They focused more on environmental, social and psychological challenges associated with music experiences, which have received limited attention in extant research.

Regarding music, although pitch perception has been a strong emphasis for many studies, these participants most frequently named music complexity and familiarity as issues influencing their music experiences; background music was described as a major impediment to satisfactory conversations. Relatively speaking, only a modest body of research has focused on these concerns to date (e.g., Gfeller et al., 2003, 2005, 2012; Eskridge et al., 2012; Başkent et al., 2014). Familiarity and complexity (including the complexity of speech and music) are interesting variables in relation to signal processing, as well as the auditory profile and cognitive processes of listeners, and present factors ripe for deeper exploration.

Highly controlled studies that focus on the underlying mechanisms of electric hearing and music are essential, and must remain a high priority for device development and basic science. However, until a more “normal” musical signal can be conveyed through electric hearing, the experiences of these CI users suggests the need for a complimentary agenda of research and clinical counseling. That agenda should more fully tap into the dynamic and multifaceted nature of music listening in real world conditions, as well as rehabilitation or accommodations for managing noisy public places or complex sounds.

This recommendation should not be viewed as an “either or” situation. The phenomenon of hearing loss and CI use is complex and multifaceted. Thus many research foci and approaches are necessary to move hearing science forward, while also optimizing real-world listening experiences for the many CI users who continue to live with past and current-generation technology.

The participants in this study highlighted psychosocial aspects of music experiences, particularly effort and coping, foci that have received scant attention in prior studies of CIs and music. The effort that these participants described in music experiences was similar to outcomes reported in recent studies of speech communication (e.g., Harris et al., 2016; Pichora-Fuller et al., 2016; Hughes et al., 2018). Listening goals often depend on the amount of cognitive energy expended, with greater energy required when the quality of the signal is poor (Pichora-Fuller et al., 2016); a degraded signal is common for many musical sounds. Situations requiring effort beyond the listener’s current capacity may be unsustainable and result in withdrawal from social experiences including music. As noted by Dritsakis et al. (2017), avoidance of music has negative implications for quality of life.

Conversely, some individuals with similar listening abilities may consider the same situations as motivational challenge and, thus, choose to persevere (Pichora-Fuller et al., 2016). Thus, clinical guidance for rehabilitation or counseling should take into account the problem-solving orientation of the individual and motivational factors that promote active listening and problem solving, such as social connection and preferred or culturally relevant music.

The prevalence of remarks from these CI users regarding effortful listening in noisy environments is a concern, not only from a communication standpoint but also in relation to physical and emotional health of CI users. According to Sarafino and Smith (2016), individuals experience greater stress when (1) circumstances are perceived as outside of one’s control; (2) when effort is accompanied by distress; (3) when social connections are undermined; (4) when the individual lacks resources; and (5) when functioning in noisy environments. These stressors were all associated with music experiences described by these participants. Chronic stressors are associated with higher prevalence of medical problems and reduced quality of life (Sarafino and Smith, 2016). Thus, the concerns expressed by these CI users have implications for general well-being and should be addressed in rehabilitation, along with hearing device optimization. This also suggests the need for public advocacy and education regarding “listener friendly” environments.

The dynamic nature of music listening as expressed by these CI users brings to mind the term, “coping,” which has been defined by some as a dynamic process in which people try and manage the perceived discrepancy between life demands and the resources they need to manage stressful situations. When coping with a difficult situation, the individual has ongoing transactions with the environment in which they are required to appraise and re-appraise the influential factors and response options. Thus coping is an on-going and ever changing process (Sarafino and Smith, 2016). This suggests that counseling CI users regarding music listening should include not only helpful “listening tips,” but should also include guidance to enhance self-efficacy, realistic expectations, flexible problem solving, and provision of access to useful information, including training programs, that can be accessed on an on-going basis.

An unexpected result of this study was the top priorities of these CI users for music training. Ninety percent indicated the desire to have training programs, but emphasized that the content should be practical, and should include personally meaningful listening tasks. Thus, analytic exercises of isolated structural components intended to enhance basic auditory processing might be complemented with tasks that recipients see as relevant to daily life.

Most training studies, to date, have focused on enhancing pitch perception, or melody and timbre recognition (see review, Looi et al., 2012). Surprisingly the musical component most frequently identified by this sample was ability to hear sung lyrics. Perception of sung lyrics against background accompaniment constitutes a speech in noise task, thus training for song lyrics could offer a challenging but relevant rehabilitative task beneficial to speech as well as music perception.

These participants varied in what content and format they desired for training, suggesting the need for a clinically useful menu of options with regard to purpose, content, and prerequisite skills. For example, particular forms of information may be more or less accessible depending upon the specific CI user based upon past music training or onset of hearing loss (e.g., reading music notation, mental representation of “normal” pitch).

Participants also emphasized that their busy lives precluded intensive time commitments for training. Most of the participants interested in training were willing to train for approximately 30 min, several times a week, over one or two weeks. Neuroplasticity requires ample repetition over time. Thus, the concern regarding time commitment brings up important questions of how much training is required for measurable improvements, and protocol components that support compliance and persistence. Individual priorities should be considered when counseling CI users on music-based training options.

The participants in our sample revealed a range of responses to music experiences from active problem solving to avoidance and withdrawal. Attitudes and behaviors toward rehabilitation are in part a function of the individual’s problem solving orientation. What factors are associated with a strong problem solving orientation? Can a problem solving orientation be encouraged or taught?

Future studies and clinical guidance may be informed by research and clinical approaches in psychology. According to cognitive psychologist Albert Bandura (2010), self-efficacy is the belief in one’s capabilities to organize and execute the course of action required to manage situations. It is determined by motivation, amount of effort extended in, how long you will persist in the face of adversity, and belief that you can ultimately succeed. These beliefs are related to one’s sense of mastery in past tasks relevant to the target goal, modeling by others, social persuasion, social support, and positive emotional state. An examination of methods for developing self-efficacy may be clinically useful avenues for future counseling of CI recipients.

The results of this study indicate a need for accessible information that audiologists can quickly and easily share with their patients and families. These participants’ comments regarding lack of input from audiologists on rehabilitation was consistent with findings by Harris et al. (2016). It is also possible that busy audiologists, who must typically focus on speech outcomes, may lack time to delve into factors influencing music perception and enjoyment. This gap between research knowledge and clinical application may reflect in part fiscal and system pressures associated with healthcare delivery. According to one study, more than 50% of audiologists have 90 min or less to take care of audiograms, speech testing, mapping and troubleshooting during audiology visits. Little if any time remains for counseling on unique challenges such as music listening (Dunn, 2018). Thus, it is of little surprise that audiologists do not provide information and guidance about music and complex listening environments.

These participants and prior studies have noted limited reimbursement for rehabilitative strategies for adult hearing device users following implantation or device acquisition. This lack of rehabilitation is notable, in that CIs can have direct costs of more than three times the cost of a knee replacement, yet the typical adult hearing device recipient in the United States often does not have access to a structured rehabilitation program (Harris et al., 2016). Professional organizations and audiologists might advocate for rehabilitation programs for adult CI recipients to optimize CI use in challenging listening conditions. In addition, these findings may suggest the need for assessment protocols that more fully document outcomes in real-world environments as well as speech perception in controlled test environments. The themes from this study may provide a foundation for the development of an assessment tool for evaluating successful outcomes in real-life conditions.

This study has limitations that should be discussed. While concerted efforts were made to recruit a diverse sample with regard to music experiences and background, it is possible that this study tended to attract CI users for whom music is personally important. The participants were primarily residents of the United States, thus their experiences are more likely consistent with health care and social practices within the United States. These enrollment patterns may limit the relevance of these results to the larger population of CI users. While inter-rater reliability and member checking were used to verify and validate the analyses, the questions and analyses were subject to the viewpoints of the authors and consultants, whose perspectives on CIs and music may not represent the range of important perspectives on this topic. Consistent with principles of qualitative methodology, this study was not intended to provide objective “truths” confirmed though testable hypotheses. Further research is needed to test the impact and applicability of the themes that emerged from this sample to other subgroups and the larger CI population.

In closing, through the voices of these CI users, we see a glimpse into the everyday music experiences and the challenges associated with using a helpful but imperfect listening device for music. Music continues to be a challenge for the many thousands of CI and HA users who will continue to rely on current technology (Gfeller et al., 2012). The Dynamic Problem Solving Model for Management of Music Listening Environments offers a useful organizational tool for considering relevant variables for research on real-life music experiences, and clinical initiatives that are consistent with the priorities of the CI users in everyday life. Possible initiatives might include experiments that more nearly reflect the dynamic and multifaceted nature of real-life listening environments, rehabilitative programs that include psychosocial factors, the development of user-friendly resources, and public advocacy for listener-friendly public spaces.

The comments from these CI users reveal that satisfactory music experiences in real life are complex in nature, and a simple “how to” list for improvement is unlikely to “fix” the problems. However, audiologists may provide helpful guidance by discussing or having at hand available links to websites or reader-friendly articles (unrelated to device marketing) that clarify why music listening is challenging, and strategies from other CI users. These resources might emphasize and explain the dynamic nature of music listening and the need for on-going problem solving. Strategies might include enrollment of one’s social network, use of ALDs for specific situations, self-directed training programs, and options for managing the listening environment. Different approaches, including rehabilitation, accommodations, judicious choice of music and listening environment, and avoidance of particularly difficult situations all have potential benefits, depending upon the dynamic combination of music, environment, and listener characteristics.

## Data Availability Statement

The datasets for this study will not be made publicly available because the study protocol was approved by the University IRB on the condition that data were not to be shared with members not a part of the research team as participants could be identified based upon their responses. They did not provide consent for such identification.

## Ethics Statement

This study was carried out in accordance with the requirements of the University of Iowa Human Subjects Institutional Review Board. All participants were provided with an invitation to participate and a letter of consent. Those who wished to participate responded to VD and informed consent was implied. This protocol was approved by the University of Iowa Human Subjects Institutional Review Board.

## Author Contributions

All authors contributed to the conceptualization of the patient-centered methodology and the development of the measurement tool. KG was primary author for the grant application that supported this project. AS had primary oversight for the development of the online focus group protocol and recruitment of participants. VD had primary oversight for first and second cycle coding and inductive analyses of the dataset. KG had primary oversight for the deductive coding and the development of the model proposed in this manuscript. KG and VD were primary authors of the manuscript, and AS contributed feedback.

## Conflict of Interest

The authors declare that the research was conducted in the absence of any commercial or financial relationships that could be construed as a potential conflict of interest.

## References

[B1] Attride-Stirling J. (2001). Thematic networks: an analytic tool for qualitative research. *Qual. Res.* 1 385–405. 10.1177/146879410100100307

[B2] Bandura A. (2010). “Self-efficacy,” in *The Corsini Encyclopedia of Psychology*, eds Weiner I. B. Craighead W. E. (Hoboken, NJ: John Wiley & Sons, Inc), 1–3.

[B3] Bartel L. R. Greenberg S. Friesen L. M. Ostroff J. Bodmer D. Shipp D. (2011). Qualitative case studies of five cochlear implant recipients’ experience with music. *Cochlear Implants Int.* 12 27–33. 10.1179/146701010x486435 21756456

[B4] Başkent D. van Engelshoven S. Galvin J. J. III (2014). Susceptibility to interference by music and speech maskers in middle-aged adults. *J. Acoust. Soc. Am.* 135 EL147–EL153. 10.1121/1.4865261 24606308 PMC4043475

[B5] Bradley E. H. Curry L. A. Devers K. J. (2007). Qualitative data analysis for health services research: developing taxonomy, themes, and theory. *Health Serv. Res.* 42 1758–1772. 10.1111/j.1475-6773.2006.00684.x 17286625 PMC1955280

[B6] Brodt M. Norton M. Christine K. Kratchman A. (2015). So much more than a “pair of brown shoes”: triumphs of patient and other stakeholder engagement in patient-centered outcomes research. *Pat. Exp. J.* 2 43–49. 10.35680/2372-0247.1057

[B7] Clancy C. Collins F. S. (2010). Patient-Centered outcomes research institute: the intersection of science and health care. *Sci. Transl. Med.* 2:37cm18. 10.1126/scitranslmed.3001235 20574065

[B8] Collister L. B. Huron D. (2008). Comparison of word intelligibility in spoken and sung phrases. *Empir. Musicol. Rev.* 3 109–125. 10.18061/1811/34102

[B9] Creswell J. W. (2014). *A Concise Introduction to Mixed Methods Research.* Thousand Oaks, CA: SAGE publications.

[B10] Domecq J. P. Prutsky G. Elraiyah T. Wang Z. Nabhan M. Shippee N. (2014). Patient engagement in research: a systematic review. *BMC Health Serv. Res.* 14:89. 10.1186/1472-6963-14-89 24568690 PMC3938901

[B11] Drennan W. R. Oleson J. J. Gfeller K. Crosson J. Driscoll V. D. Won J. H. (2015). Clinical evaluation of music perception, appraisal and experience in cochlear implant users. *Int. J. Audiol.* 54 114–123. 10.3109/14992027.2014.948219 25177899 PMC4297259

[B12] Dritsakis G. van Besouw R. M. O’Meara A. (2017). Impact of music on the quality of life of cochlear implant users: a focus group study. *Cochlear Implants Int.* 18 207–215. 10.1080/14670100.2017.1303892 28326996

[B13] Dunn C. (2018). “Post-implant immediate and long-term care,” in *CI2018 Emerging Issues in Cochlear Implantation* (Washington, DC: American Cochlear Implant Alliance).

[B14] Eskridge E. N. Galvin J. J. III Aronoff J. M. Li T. Fu Q. J. (2012). Speech perception with music maskers by cochlear implant users and normal-hearing listeners. *J. Speech Lang. Hear Res.* 55 800–810. 10.1044/1092-4388(2011/11-0124) 22223890 PMC5847337

[B15] Forsythe L. P. Frank L. B. Workman T. A. Hilliard T. Harwell D. Fayish L. (2017). Patient, caregiver and clinician views on engagement in comparative effectiveness research. *J. Comp. Effect. Res.* 6 231–244. 10.2217/cer-2016-0062 28173732

[B16] Frank L. Basch E. Selby J. V. (2014). The PCORI perspective on patient-centered outcomes research. *JAMA* 312 1513–1514. 25167382 10.1001/jama.2014.11100

[B17] Fujita S. Ito J. (1999). Ability of nucleus cochlear implantees to recognize music. *Ann. Otol. Rhinol. Laryngol.* 108(7 Pt 1), 634–640. 10.1177/000348949910800702 10435919

[B18] Fuller C. D. Galvin J. J. III Maat B. Başkent D. Free R. H. (2018). Comparison of two music training approaches on music and speech perception in cochlear implant users. *Trends Hear.* 22 1–22. 10.1177/2331216518765379 29621947 PMC5894911

[B19] Gfeller K. Buzzell A. Driscoll V. Kinnaird B. Oleson J. (2009). “The impact of voice range and instrumental background accompaniment on recognition of song lyrics,” in *Proceedings of the 7th Asia Pacific Symposium on Cochlear Implants and Related Sciences*, Singapore, 35–40.

[B20] Gfeller K. Christ A. Knutson J. Witt S. Mehr M. (2003). The effects of familiarity and complexity on appraisal of complex songs by cochlear implant recipients and normal hearing adults. *J. Music Ther.* 40 78–112. 10.1093/jmt/40.2.78 14505444

[B21] Gfeller K. Christ A. Knutson J. F. Witt S. Murray K. T. Tyler R. S. (2000). Musical backgrounds, listening habits, and aesthetic enjoyment of adult cochlear implant recipients. *J. Am. Acad. Audiol.* 11 390–406. 10976500

[B22] Gfeller K. Jiang D. Oleson J. J. Driscoll V. Knutson J. F. (2010). Temporal stability of music perception and appraisal scores of adult cochlear implant recipients. *J. Am. Acad. Audiol.* 21 28–34. 10.3766/jaaa.21.1.4 20085197 PMC2844251

[B23] Gfeller K. Lansing C. (1992). Musical perception of cochlear implant users as measured by the primary measures of music audiation: an item analysis. *J. Music Ther.* 29 18–39. 10.1093/jmt/29.1.18

[B24] Gfeller K. Lansing C. R. (1991). Melodic, rhythmic, and timbral perception of adult cochlear implant users. *J. Speech Hear. Res.* 34 916–920. 10.1044/jshr.3404.916 1956198

[B25] Gfeller K. Oleson J. Knutson J. F. Breheny P. Driscoll V. Olszewski C. (2008). Multivariate predictors of music perception and appraisal by adult cochlear implant users. *J. Am. Acad. Audiol.* 19 120–134. 10.3766/jaaa.19.2.3 18669126 PMC2677551

[B26] Gfeller K. Olszewski C. Rychener M. Sena K. Knutson J. F. Witt S. (2005). Recognition of “real-world” musical excerpts by cochlear implant recipients and normal-hearing adults. *Ear Hear.* 26 237–250. 10.1097/00003446-200506000-00001 15937406

[B27] Gfeller K. Turner C. Mehr M. Woodworth G. Fearn R. Knutson J. F. (2002). Recognition of familiar melodies by adult cochlear implant recipients and normal-hearing adults. *Cochlear Implants Int.* 3 29–53. 10.1179/cim.2002.3.1.29 18792110

[B28] Gfeller K. Turner C. Oleson J. Kliethermes S. Driscoll V. (2012). Accuracy of cochlear implant recipients in speech reception in the presence of background music. *Ann. Otol. Rhinol. Laryngol.* 121 782–791. 10.1177/000348941212101203 23342550 PMC3686524

[B29] Gregory M. (2011). Tools for enabling communication partners. *Hear. J.* 64 44–46.

[B30] Hargreaves D. J. North A. C. (2010). “Experimental aesthetics and liking for music,” in *Handbook of Music and Emotion: Theory, Research, Applications*, eds Juslin P. N. Sloboda J. A. (Oxford: Oxford University Press), 515–546. 10.1093/acprof:oso/9780199230143.003.0019

[B31] Harris M. S. Capretta N. R. Henning S. C. Feeney L. Pitt M. A. Moberly A. C. (2016). Postoperative rehabilitation strategies used by adults with cochlear implants: a pilot study. *Laryngoscope Investig. Otolaryngol.* 1 42–48. 10.1002/lio2.20 28894803 PMC5510267

[B32] Hickam D. Totten A. Berg A. Rader K. Goodman S. Newhouse R. (2013). *The PCORI Methodology Report.* Washington: PCORI.

[B33] Hill-Briggs F. (2003). Problem solving in diabetes self-management: a model of chronic illness self-management behavior. *Ann. Behav. Med.* 25 182–193. 10.1207/s15324796abm2503_04 12763713

[B34] Hughes S. E. Hutchings H. A. Rapport F. L. McMahon C. M. Boisvert I. (2018). Social connectedness and perceived listening effort in adult cochlear implant users: a grounded theory to establish content validity for a new patient-reported outcome measure. *Ear Hear.* 39 922–934. 10.1097/AUD.0000000000000553 29424766

[B35] Krueger R. Casey M. (2009). *Focus Groups: A Practical Guide to Applied Science.* Thousand Oaks, CA: Sage.

[B36] Lassaletta L. Castro A. Bastarrica M. Perez-Mora R. Herran B. Sanz L. (2008). Changes in listening habits and quality of musical sound after cochlear implantation. *Otolaryngol. Head Neck Surg.* 138 363–367. 10.1016/j.otohns.2007.11.028 18312886

[B37] Limb C. J. Roy A. T. (2014). Technological, biological, and acoustical constraints to music perception in cochlear implant users. *Hear. Res.* 308 13–26. 10.1016/j.heares.2013.04.009 23665130

[B38] Looi V. Gfeller K. Driscoll V. (2012). Music appreciation and training for cochlear implant recipients: a review. *Semin. Hear.* 33 307–334. 10.1055/s-0032-1329222 23459244 PMC3583543

[B39] Looi V. She J. (2010). Music perception of cochlear implant users: a questionnaire, and its implications for a music training program. *Int. J. Audiol.* 49 116–128. 10.3109/14992020903405987 20151886

[B40] Mather M. Hamilton D. Robalino S. Rousseau N. (2018). Going where other methods cannot: a systematic mapping review of 25 years of qualitative research in Otolaryngology. *Clin. Otolaryngol.* 43 1443–1453. 10.1111/coa.13200 30062706

[B41] Migirov L. Kronenberg J. Henkin Y. (2009). Self-reported listening habits and enjoyment of music among adult cochlear implant recipients. *Ann. Otol. Rhinol. Laryngol.* 118 350–355. 10.1177/000348940911800506 19548384

[B42] Miles M. B. Huberman A. M. Saldaña J. (2014). *Qualitative Data Analysis: A Methods Sourcebook.* Thousand Oaks, CA: SAGE Publications.

[B43] Mirza S. Douglas S. A. Lindsey P. Hildreth T. Hawthorne M. (2003). Appreciation of music in adult patients with cochlear implants: a patient questionnaire. *Cochlear Implants Int.* 4 85–95. 10.1179/cim.2003.4.2.85 18792140

[B44] Philips B. Vinck B. De Vel E. Maes L. D’Haenens W. Keppler H. (2012). Characteristics and determinants of music appreciation in adult CI users. *Eur. Arch. Otorhinolaryngol.* 269 813–821. 10.1007/s00405-011-1718-1714 21847672

[B45] Pichora-Fuller M. K. Kramer S. E. Eckert M. A. Edwards B. Hornsby B. W. Humes L. E. (2016). Hearing impairment and cognitive energy: the framework for understanding effortful listening (FUEL). *Ear Hear.* 37 5S–27S. 10.1097/aud.0000000000000312 27355771

[B46] Pijl S. (1995). Musical pitch perception with pulsatile stimulation of single electrodes in patients implanted with the Nucleus cochlear implant. *Ann. Otol. Rhinol. Laryngol. Suppl.* 166 224–227. 7668647

[B47] Pisoni D. B. Kronenberger W. G. Harris M. S. Moberly A. C. (2017). Three challenges for future research on cochlear implants. *World J. Otorhinolaryngol. Head Neck Surg.* 3 240–254. 10.1016/j.wjorl.2017.12.010 29780970 PMC5956139

[B48] Plant G. Bernstein C. M. Levitt H. (2015). Optimizing performance in adult cochlear implant users through clinician directed auditory training. *Semin. Hear.* 36 296–310. 10.1055/s-0035-1564460 27587916 PMC4910544

[B49] Saldaña J. (2013). *The Coding Manual for Qualitative Researchers.* London: Sage.

[B50] Sarafino E. P. Smith T. W. (2016). *Health Psychology: Biopsychosocial Interactions*, 9th Edn Hoboken, NJ: John Wiley & Sons.

[B51] Savenye W. C. Robinson R. S. (1996). “Qualitative research issues and methods: an introduction for educational technologists,” in *Handbook of Research for Educational Communications and Technology*, eds Jonassen D. H. Harris P. (Mahwah, NJ: Lawrence Erlbaum Associates), 1171–1195.

[B52] Sheridan S. Schrandt S. Forsythe L. Hilliard T. S. Paez K. A. (2017). The PCORI engagement rubric: promising practices for partnering in research. *Ann. Fam. Med.* 15 165–170. 10.1370/afm.2042 28289118 PMC5348236

[B53] Sloboda J. A. (2010). “Music in everyday life: the role of emotions,” in *Handbook of Music and Emotion: Theory, Research, Applications*, eds Juslin P. N. Sloboda J. A. (New York, NY: Oxford University Press), 493–514.

[B54] Smith S. L. (2014). Promoting self-efficacy in patient-centered audiologic rehabilitation for adults with hearing loss. *Perspect. Aural Rehabil. Instrum.* 21 24–32.

[B55] Smith S. L. West R. L. (2006). The application of self-efficacy principles to audiologic rehabilitation: a tutorial. *Am. J. Audiol.* 15 46–56. 10.1044/1059-0889(2006/006) 16803791

[B56] Strauss A. Corbin J. (1994). Grounded theory methodology. *Handb. Qual. Res.* 17 273–285.

[B57] Tates K. Zwaanswijk M. Otten R. van Dulmen S. Hoogerbrugge P. M. Kamps W. A. (2009). Online focus groups as a tool to collect data in hard-to-include populations: examples from paediatric oncology. *BMC Med. Res. Methodol.* 9:15. 10.1186/1471-2288-9-15 19257883 PMC2653071

[B58] Wilhelm L. A. (2016). *Accessibility of Music Experiences for Individuals With Age-Related Hearing Loss.* Ph.D.thesis, The University of Iowa, Iowa.

[B59] Wright R. Uchanski R. M. (2012). Music perception and appraisal: cochlear implant users and simulated cochlear implant listening. *J. Am. Acad. Audiol.* 23 350–365; quiz379. 10.3766/jaaa.23.5.6 22533978 PMC3400338

[B60] Young-Hyman D. De Groot M. Hill-Briggs F. Gonzalez J. S. Hood K. Peyrot M. (2016). Psychosocial care for people with diabetes: a position statement of the American Diabetes Association. *Diabetes Care* 39 2126–2140. 27879358 10.2337/dc16-2053 PMC5127231

