# Supplementary material for: Adult Cochlear Implant Recipients’ Perspectives on Experiences With Music in Everyday Life: A Multifaceted and Dynamic Phenomenon
Source: Front Neurosci. 2019 Nov 21;13:1229. doi: 10.3389/fnins.2019.01229 (PMC6882382; doi:10.3389/fnins.2019.01229)
Supplement: Supplementary file 1 [file Table_1.DOCX]

Appendix A. Questionnaire instructions and items

| **Instructions** | |
| --- | --- |
|  | We are interested in your experiences of complex listening situations including those with music in everyday life as a CI user. This includes several types of experiences:  1) Purposeful listening to music, such as in concerts, in spiritual settings, or on your stereo or radio.  2) Background music that competes with speech, such as background music in restaurants or at social events, or music behind the dialogue of movies and TV shows.  As you answer the following questions, please keep in mind that we would like to learn about specific types of sounds or situations that represent easy and difficult listening. Through your input, we hope to better understand the needs and priorities of CI users when it comes to real-life experiences with music and speech.  The questions will focus on 4 areas: 1) Purposeful listening to music, such as in concerts, in spiritual settings, or on your stereo or radio; 2) spoken conversations with background music, 3) training programs, and 4) support systems for CI users.  We have chosen to use open-ended questions with the hope that we can get a more detailed picture of YOUR unique life experiences, whether good, bad, or indifferent.  You are free to skip any questions you prefer not to answer, or for which you have no relevant experiences to share. |
| **Listening to Music** | |
| 1. | How much did you enjoy listening to music before hearing loss and implantation? |
| 2. | Describe your experience with music after getting your CI. Is it effortful or difficult? Or is it easy to enjoy? |
| 3. | How would you describe the sound quality of music through your CI? |
| 4. | If you use a hearing aid as well as CI, does using the hearing aid affect the sound quality of music? |
| 5. | What assistive devices (for example, external microphone, ADRO, Whisper, etc.), if any, have helped you to function in complex listening situations with music? |
| 6. | What technical features (music program?) of the CI, if any, have helped you during complex listening situations with music ? |
| 7. | Are some kinds of music (familiar vs. unfamiliar, specific styles, etc.) easier to enjoy or understand than others? |
| 8. | Are some listening environments (live concerts, on the stereo, etc.) better for music listening than others? |
| 9. | What accommodations, if any, have you used to improve your ability to function in complex listening situations with music? |
| 10. | What sorts of complex listening situations with music do you experience in social situations? Have you had to modify your social life as a result (for example, avoid some situations, or use specific strategies)? If applicable, why have you had to make such modifications? Have they helped? |
| **Speech against competing music.** | |
| 11. | Please describe your experiences of background music in social conversational settings such as parties or restaurants. |
| 12. | Please describe your experiences when listening to the spoken dialogues against background music in movies, videos, or on TV. |
| 13. | What assistive devices (for example, external microphone, ADRO, Whisper, etc.), if any, have helped you to function with speech in background music? |
| 14. | What technical features (special signal processing programs, directional microphones?) of the CI, if any, have helped you during speech in background music? |
| **Music Training** | |
| 15. | What if any aspects of music listening would you like to improve?  (for example, being able to recognize melodies,  being able to pick out the lyrics in songs? Other?) |
| 16. | If you could have a training program tailor made for you, what would it include (for example, types of music, format)? |
| 17. | Are you aware of rehabilitation training programs for music? Have you used any of these sources, and if so, which ones? |
| 18. | If you chose not to use available rehabilitation, why not? |
| 19. | If you are interested in training or rehabilitation sources for music, are there barriers to using those resources (for example, lack of availability, lack of insurance coverage, too much time commitment, overly boring, or cost)? |
| 20. | How comfortable would you feel using a technology-based training program, like one that is online or computer based? Would you prefer a program with a social component? |
| 21. | What is reasonable or realistic for training programs/rehabilitation: 1. How much money? 2. How much time? 3. Other factors that would encourage you to use rehabilitation resources? |
| **Support Systems** | |
| 22. | During regular audiology appointments, have you ever discussed rehabilitation or strategies for coping with complex listening situations? If not, why (not interested, not enough time, insurance doesn’t cover counseling beyond testing and mapping, etc.)? |
| 23. | Has your audiologist ever given practical suggestions or resources to improve complex listening (speech in background noise or music; music listening)? |
| 24. | Has your audiologist helped you find or navigate sources for improving music listening? If so, how? |
| 25. | What resources (support or advocacy groups, on-line blogs and web sites, informal conversations with other CI users, etc.) if any, have worked for you to improve complex listening situations such as music listening, speech in background music or noise, etc.? |
| Other | |
| 26. | You are an expert on living with a CI. Are there any questions about complex listening situations that we did not ask but that you feel we should be aware of? |
| 27. | Please include any other comments here. |

Thanks so very much for your insights, and for sharing your experiences with us.
