# Supplementary material for: Adult Cochlear Implant Recipients’ Perspectives on Experiences With Music in Everyday Life: A Multifaceted and Dynamic Phenomenon
Source: Front Neurosci. 2019 Nov 21;13:1229. doi: 10.3389/fnins.2019.01229 (PMC6882382; doi:10.3389/fnins.2019.01229)
Supplement: Supplementary file 2 [file Table_2.DOCX]

Appendix B. Magnitude coding of themes, codes and subcodes with frequency of responses in number and proportion. The right hand column presents the definitions used in the coding process.

| 1. Strategies (all 40 respondents) | Adaptations made by listener to successfully navigate complex listening environments |
| --- | --- |
| 1.1 Technology (37/40, 92.5%) | Items used as supplement to cochlear implants |
| 1.1.1 Tools (32/40, 80%) | Accessories used in combination with cochlear implant |
| 1.1.2 Settings/Programs (25/40, 62.5%) | Programming strategies used such as ADRO, Whisper, or SCAN, sometimes also referred to as P# |
| 1.1.3 Closed Captioning (23/40, 57.5%) | The process of displaying text on a screen or other visual display to provide additional information regarding spoken dialogue |
| 1.1.4 Adjust volume/sensitivity (11/40, 27.5%) | The adjustment of specific parameters on the processor, while not changing the programming strategy |
| 1.1.5 Remove CI (7/40, 17.5%) | Turning off and/or removal of external processor |
| 1.2 Individual Characteristics (37/40, 92.5%) | Traits that establish a uniqueness that can be linked to successful experience |
| 1.2.1 Assertiveness/Advocacy (24/40, 60%) | Behavior that modifies others’ interactions for subject benefit |
| 1.2.2 Effort/Active Listening (21/40, 52.5%) | Energy put forth toward successful listening |
| 1.2.3 Lip-reading (13/40, 32.5%) | The act of watching the face or lip movement to understand spoken words/language |
| 1.2.4 Control Environment/Music (9/40, 22.5%) | Active choice of volume or music to achieve music listening success |
| 1.2.5 Guess/Fill-in/Make-up  Answer/Passive Listening (6/40, 15%) | Attempts to understand with minimal effort, giving up, resigning to lack of success |
| 1.3 Music (29/40, 72.5%) | Overarching definition related to components for music elements |
| 1.3.1 Repetition/Practice (22/40, 55%) | Repeated listening or use of rehabilitation programs designed to improve listening/perception |
| 1.3.2 Familiarity (16/40, 40%) | Use/selection of music known and/or enjoyed previously |
| 1.3.3 Genre/Choice (7/40, 17.5%) | Selection of specific styles of music that appeal to user |
| 1.3.4 Look up lyrics (8/40, 20%) | Finding words to music to compare understanding or ensure understanding |
| 1.4 Environment (28/40, 70%) | Overarching choice of listening area/space |
| 1.4.1 Location/Proximity (23/40, 57.5%) | Distance to/from a sound source or acoustic environment |
| 1.4.2 Conditions (20/40, 50%) | Selection of space based upon acoustics of an area that are pleasant |
| 1.5 Support (22/40, 55%) | Intangible assistance given to the listener that benefits their experience |
| 1.5.1 Other CI recipients (15/40, 37.5%) | Information from individuals with cochlear implants |
| 1.5.2 Friends/Family (11/40, 27.5%) | Information or understanding from friends, family, and clinical professionals (i.e., audiologists) |
| 1.5.3 Information (10/40, 25%) | Websites, blogs, and other social media that provide guidance or suggestions for positive listening experiences |
| 1.6 Avoid (29/40, 72.5%) | Locations, situations, social events in which a listening intentionally keeps themselves away |
| 1.6.1 Situation (21/40, 52.5%) | Events, gatherings, locations that a recipients would actively choose to not attend or participate |
| 1.6.2 Sound Source (15/40, 37.5%) | A point of origin for sound that is identifiable and able to be located |
| 2. Barriers (all 40 respondents) | Experiences, environments, and situations that inhibit successful listening |
| 2.1 Environment (36/40, 90%) | Overarching choice of listening area/space |
| 2.1.1 Competing sounds/Background (31/40, 77.5%) | Additional sounds or music added to an environment that inhibit accurate or enjoyable perception |
| 2.1.2 Location (30/40, 75%) | Venues that are known for persisting in background sounds |
| 2.1.3 Other people (9/40, 22.5%) | Additional voices added to environment in addition to the target sound source |
| 2.2 Music Properties (34/40, 85%) | Components which inhibit music perception/enjoyment independently or in combination |
| 2.2.1 Complexity (27/40, 67.5%) | The quality or condition with multiple instruments/players or types of instruments |
| 2.2.2 Loudness (19/40, 47.5%) | Amplitude related to inhibition of music enjoyment |
| 2.2.3 Familiarity (15/40, 37.5%) | Lack of experience with specific music styles, genres, or groups that inhibit enjoyment |
| 2.2.4 Pitch (7/40, 17.5%) | Frequency range related to musical instruments and vocalists that lead to a more difficult perception/enjoyment |
| 2.3 User Characteristics (30/40, 75%) | Individual differences that differentiate listeners’ success |
| 2.3.1 Effort (25/40, 62.5%) | Energy put forth by the listener required for successful experience |
| 2.3.2 Personal characteristics (12/40, 30%) | Individual differences between CI users that include personality, unique experiences, that affect listening experiences |
| 2.4 Access (33/40, 82.5%) | Availability of information and expenses related to rehabilitation tools, strategies and support |
| 2.4.1 Knowledge (26/40, 65%) | Knowledge or availability of information, technology, expertise that could potentially assist in enjoyment |
| 2.4.2 Financial (23/40, 57.5%) | Cost related to use, maintenance, and upgrade of elements of the device or rehabilitation |
| 2.5 Technology (26/40, 65%) | Items intended as supplement to cochlear implants that are not perceived as effective or functional |
| 2.6 Listening Method (7/40, 17.5%) | Manner in which the sound is presented that can affect quality of sound (speakers, cell phones, television, specific ear/device) |
| 3. Pleasant Experience (39 respondents) | Description of sounds, experiences, and feelings of a positive connotation |
| 3.1 Music Characteristics (33/39, 84.62%) | Overarching definition related to components for music elements |
| 3.1.1 Complexity (21/39, 53.85%) | the quality or condition with multiple instruments/players or types of instruments |
| 3.1.2 Familiarity (21/39, 53.85%) | Use/selection of music known and/or enjoyed previously |
| 3.1.3 Pitch (6/39, 15.38%) | Frequency range related to musical instruments and vocalists that lead to perception/enjoyment |
| 3.1.4 Loudness/Volume (4/39, 10.26%) | Amplitude related to music enjoyment |
| 3.1.5Timbre (3/39, 7.7%) | Tone quality related to specific instruments or voices that promote enjoyment |
| 3.2 Enjoyable (22/39, 56.41%) | Quality of sound and experience that lead to pleasant encounter. May also include the word “enjoy” |
| 3.3 Environment (23/39, 58.97%) | Overarching choice of listening area/space |
| 3.3.1 Location (20/39, 51.28%) | Venues that are known for background sound quality |
| 3.3.2 Competing/Background (8/39, 20.51%) | Environment quality selected by listeners for successful experiences (an absence of extraneous sounds) |
| 3.4 Listening Method (11/39, 28.21%) | Manner in which a listener chooses to engage in music that may include specific device configurations, locations or listening methods (i.e., stereo system, car system, etc) |
| 3.5 Technology (11/39, 28.21%) | Items used as supplement to cochlear implants |
| 3.6 Personal Characteristics (9/39, 23.08%) | Individual differences between CI users that include personality, unique experiences, that affect listening experiences |
| 3.6.1 Perseverance (7/39, 17.95%) | Sustaining of music listening and exposure |
| 3.6.2 Effort (3/39, 7.69%) | Ability to enjoy music with minimal focus or attention |
| 4. Unpleasant Experience (33 respondents) | Description of sounds, experiences, and feelings of a negative connotation |
| 4.1 Environment (23/33, 69.70%) | Overarching effect of listening area/space |
| 4.1.1 Location (20/33, 60.61%) | Specific venues or situations that affect listening experiences |
| 4.1.2 Competing background (16/33, 48.48%) | Additional noise, music, conversations that interfere or create challenging listening environment |
| 4.2 Sound Quality (22/33, 66.67%) | Description of auditory signal as it relates to enjoyment |
| 4.3 Music (18/33, 54.55%) | Overarching definition related to components for music elements |
| 4.3.1 Complexity (10/33, 30.30%) | the quality or condition with multiple instruments/players or types of instruments |
| 4.3.2 Loud (9/33, 27.27%) | Amplitude related to music enjoyment |
| 4.3.3 Familiarity (5/33, 15.15%) | Use/selection of music not previously known and/or enjoyed |
| 4.4 Technology (8/33, 24.24%) | Items used as supplement to cochlear implants |
| 4.5 Emotional Response (10/33, 30.30%) | Emotions or reactions expressed in regards to challenging situations |
| 5. Desires (38/40) | Improvements with music and/or speech in background noise currently unavailable |
| 5.1 Training Method (34/38, 89.47%) | Method of delivery for training |
| 5.1.1 Computer/Online (28/38, 73.68%) | Computerized or available online for individuals who wish not to travel |
| 5.1.2 Time (18/38, 47.37%) | Both duration and length of lessons |
| 5.1.3 Cost (11/38, 28.95%) | Expense related to access (most prefer “Free”) |
| 5.1.4 Social (9/38, 23.68%) | A component of the program that may include interacting with others |
| 5.1.5 Appeal (8/38, 21.05%) | Factor related to willingness to participate. “Does it appeal to my interest” |
| 5.2 Music (34/38, 89.47%) | Overarching definition related to components for music elements |
| 5.2.1 Lyrics (24/38, 63.16%) | Ability to understand lyrics in music (overwhelmingly requested subjects) |
| 5.2.2 Genre (21/38, 55.26%) | Specific genres of interest: country, pop, rock, etc. as it related to each respondent |
| 5.2.3 Complexity (12/38, 31.58%) | the quality or condition with multiple instruments/players or types of instruments |
| 5.2.4 Melody Recognition (8/38, 21.05%) | The ability to determine a melody by pitch, rhythm only (absent lyrics) |
| 5.2.5 Pitch (in tune) (5/38, 13.16%) | Improving the perception of pitch via cochlear implant |
| 5.3 Hearing (11/38, 28.95%) | A desire to perceive sound as close as possible to “normal” hearing |
| 5.3.1 Support (6/38, 15.79%) | Increased personal time for audiological concerns that can be individualized to needs |
| 5.3.2 Clarity (4/38, 10.53%) | Improved discrimination and accuracy in perception |
| 5.3.3 Binaural (3/38, 7.89%) | Increased support for incorporating acoustic and electric hearing (particularly for single-sided deafness) |
| 5.4 Device (11/38, 28.95%) | Concerns related to both internal and external components of the device and its upkeep |
| 5.4.1 Cost (8/38, 21.05%) | Desires related to expenses for components of cochlear implant and its coverage |
| 5.4.2 Functional (3/38, 7.89%) | Concerns related to functionality of elements of the device and accessories |
